# Ruptured Spinal Arteriovenous Malformation: A Rare Cause of Paraplegia in Pregnancy

**DOI:** 10.1155/2018/6096483

**Published:** 2018-08-09

**Authors:** Clare E. Thiele

**Affiliations:** Royal Brisbane and Women's Hospital, Cnr Butterfield St. and Bowen Bridge Rd, Herston QLD 4029, Australia

## Abstract

**Background:**

Ruptured spinal arteriovenous malformation (AVM) is a rare cause of paraplegia in pregnancy, with only a few case reports describing complications from spinal AVMs during pregnancy in the literature.

**Case:**

A 32-year-old woman presented at 37 weeks gestation with back pain and rapidly progressive lower limb neurological symptoms. MRI showed a previously undiagnosed spinal AVM at T8. A healthy girl was delivered by caesarean under general anaesthesia to facilitate further investigation. After spinal angiography, it was concluded the most likely aetiology was acute rupture of an intra- and perimedullary AVM with associated haemorrhage at T8 secondary to venous compression from the enlarged uterus at L5 causing high pressure within the AVM and subsequent rupture. The neurosurgical and interventional radiology teams felt the lesion was not amenable to surgical or endovascular intervention. The patient remained paraplegic with no sign of neurological recovery six months after delivery.

**Conclusion:**

While new onset paraplegia during pregnancy secondary to ruptured spinal AVM is very rare, it is important to discuss these cases to inform future practice. In contrast to previous case reports, our patient did not spontaneously recover after delivery and was not amenable to surgical or endovascular treatment.

## 1. Introduction

Spinal vascular malformations, including arteriovenous malformations (AVMs), are rare. Prompt diagnosis and treatment may prevent long-term neurological disability [1–3]. Patients with spinal AVMs usually present with back pain and progressive myelopathy with gait disturbance, sensory changes, and bladder or bowel symptoms [1–3]. Proposed mechanisms for neurological deterioration include haemorrhage, redistribution of blood supply (“steal phenomena”), mass effect, or venous congestion [1, 4]. Treatment options include embolisation, surgery, combined embolisation and surgery, or conservative management depending on the specific lesion [1, 3, 4].

Ruptured spinal AVM is a rare cause of paraplegia in pregnancy, with only a few case reports describing complications from spinal vascular malformations during pregnancy in the literature [5–7]. The physiological changes of pregnancy as well as compression of venous outflow by the gravid uterus make pregnant women particularly susceptible to complications of spinal vascular malformations, precipitating venous congestion or rupture and subsequent neurological symptoms. This case describes a pregnant woman with rapidly progressive paraplegia secondary to a previously undiagnosed spinal AVM at the eighth thoracic (T8) level that ruptured during late pregnancy.

## 2. Case Presentation

A 32-year-old woman in her first pregnancy presented at 37 weeks gestation to the obstetric review centre in the late evening with a two-hour history of new onset right-sided leg pain and numbness. She was able to mobilise short distances and was otherwise well. Initially her symptoms were most suggestive of sciatica, a common complaint during pregnancy.

Her symptoms progressed rapidly over the next two hours and she reported bilateral lower limb numbness and severe shooting midthoracic back pain and was unable to move her legs. Initially she had no urinary retention or faecal incontinence. She also reported no history of trauma or any similar symptoms in the past.

She had an otherwise low risk pregnancy and there were no signs of fetal distress on arrival. Her past medical history included asthma, allergic rhinoconjunctivitis, and depression. She was a smoker and migrated to Australia from England several years earlier.

On initial examination, vital signs were normal. She was afebrile. Cardiotocograph revealed no concerns for fetal wellbeing. Her neurological examination was inconsistent but nevertheless concerning. She was found to have patchy bilateral sensory loss up to a sensory level of T10. Lower limb examination revealed reduced power bilaterally (1-2/5) across all myotomes with hyperreflexia, clonus, and upgoing plantar reflexes. Upper limb neurological examination was normal. There was no bony tenderness on palpation of her spine. Insertion of a urinary catheter five hours after presentation drained 700 ml of urine. This was suggestive of urinary retention, particularly in the context of her advanced gestation. However, she reported normal perineal sensation on catheter insertion, again inconsistent with her other symptoms and examination findings.

Due to her pregnant state, an urgent CT was not performed. An after hours MRI was not considered necessary as it was felt an acute surgical cause for the presenting signs and symptoms was unlikely. A kidney ultrasound ruled out renal stones as a cause for severe back pain.

**Figure 1:**
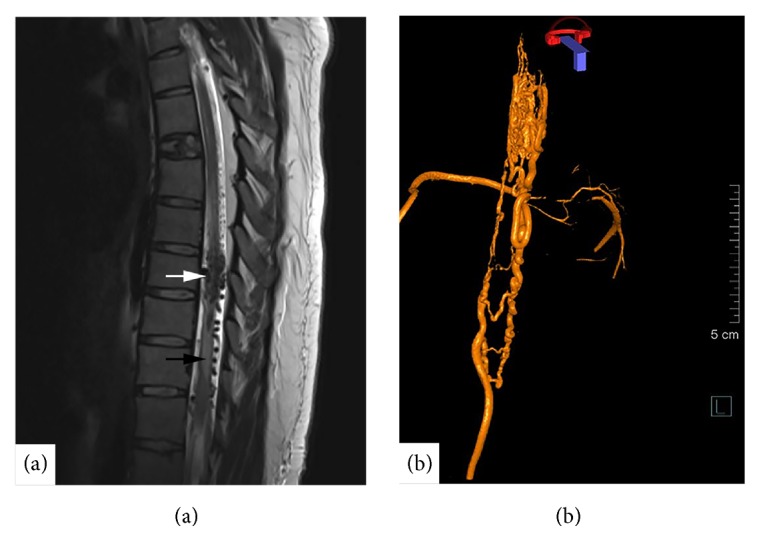
(a) Lateral T2-weighted MRI thoracic spine showing peri- and intramedullary spinal AVM at T8 (white arrow) with surrounding spinal cord oedema T6-T11 (black arrow). (b) Left lateral three-dimensional rotational spinal angiography (3D-RSA) from right T9 intercostal artery showing spinal cord AVM.

The next morning an MRI spine was performed. This revealed a previously undiagnosed mixed intra- and perimedullary spinal cord AVM at T8 with surrounding spinal cord oedema from T6-T11 (see Figure 1(a)). Her case was discussed with the neurosurgical team who felt she was not amenable to urgent surgical decompression or intervention based on MRI findings. A decision was made for urgent delivery to facilitate further investigation. A healthy baby girl was delivered that afternoon via caesarean under general anaesthetic. This was performed without complications.

Subsequent angiography showed a predominantly perimedullary slow flow spinal cord AVM with intramedullary extension at T8 to a compact nidus (see Figure 1(b)). The AVM received arterial supply from the radicular branches of the right T9 intercostal artery with a branch to the anterior spinal artery from the same level. The venous drainage of the AVM was via a single caudal draining vein that extended to the left internal iliac vein with attenuation at L5/S1.

In discussion between the radiology and neurosurgical teams, it was concluded the most likely aetiology for the patient's presentation was acute rupture of the AVM at T8 secondary to venous outflow compression from the enlarged uterus onto the draining vein at the level of L5 causing high pressure within the AVM and subsequent rupture. Given the lesion was partially within the spinal cord, treatment with surgical resection would risk potential permanent paraplegia. Additionally, she was considered not a good candidate for embolisation. As such, the patient was managed conservatively in the hope that she might have at least partial recovery of her neurological function. An inferior vena cava (IVC) filter was inserted at the time of initial angiography to prevent pulmonary emboli given the relative risk of anticoagulation in the setting of recent caesarean section and recent AVM rupture.

One month after admission, the patient developed left leg swelling and was diagnosed with a left leg extensive occlusive deep vein thrombosis extending to left external and common iliac vein as far as the IVC filter. There was concern about potential obstruction of venous outflow from the AVM precipitating further rupture as well as potential clot propagation above the IVC filter, so a decision was made for mechanical thrombectomy and removal of IVC filter. She was therapeutically anticoagulated on warfarin with clexane bridging and clot progression was monitored on weekly ultrasound scans.

Given the difficulties in finding a suitable discharge destination with a newborn baby, the patient's first few months of rehabilitation were as an inpatient in a private room on the neurosurgery ward. At the time of writing this article (six months after delivery), the patient remains paraplegic to the level of T8 with urinary and bowel incontinence. At this stage, she has a guarded prognosis for recovery.

## 3. Discussion

Cases reported in the literature of complications from spinal AVMs in pregnancy are summarised below as well as a discussion of the unique challenges of investigating and managing a pregnant woman with new onset paraplegia secondary to a spinal AVM.

Demir and colleagues published a case in* Spine* (2012) describing acute onset T8 paraplegia at 30 weeks gestation secondary to vascular congestion without rupture of a spinal vascular malformation in the setting of known Klippel-Trenaunay syndrome [5]. The patient was delivered by caesarean and her neurological symptoms improved within days of delivery without any neurosurgical intervention.

A Japanese study by Kinoshita and colleagues published in 2009 described a young woman with a known spinal vascular tumour at T2-T3 diagnosed at age 10 years [6]. She presented at 29 weeks gestation unable to walk, with progressive symptoms over the next five weeks prompting delivery via caesarean at 34 weeks gestation. Spinal angiography identified a large extradural arteriovenous fistula compressing the spinal cord at T3-T4. She was planned for endovascular embolisation but her neurological symptoms improved after delivery. She was managed conservatively and was able to walk six months after delivery.

An older study published in 1976 by Manabe and colleagues described a woman presenting at term with sudden chest pain, paraplegia, sensory level at T5, and urinary incontinence [7]. She had her delivery by caesarean and subsequently diagnosed with a typical AVM at T2-T6 on spinal angiography. Six weeks after delivery, her AVM was surgically removed and her neurological symptoms resolved.

The aforementioned studies from the literature and the one presented in this study highlight the challenges when investigating and managing paraplegia secondary to spinal vascular malformations in pregnancy.

Diagnosing spinal cord pathology in pregnancy has unique challenges. If a nonpregnant patient presented to emergency with rapidly progressive weakness and numbness suggestive of a spinal cord lesion, they would receive an urgent CT spine to rule out compressive lesions that require surgical intervention. However, radiation, especially to the abdomen, is generally avoided in pregnancy. While conventional MRI is often the first imaging performed for suspected spinal vascular malformations, it is not always readily available and is not the gold standard test. Despite advanced imaging techniques, such as time-resolved contrast-enhanced MR angiography, invasive spinal angiography is still the definitive test [1, 8]. In our patient as well as other cases in the literature, delivery of the pregnancy was necessitated to allow invasive angiography to be performed to define the lesion and determine management.

The safest choice of anaesthetic for caesarean in pregnant patients with a spinal vascular malformation requires careful consideration. A study published in 1996 by Ong et al. discussed the relative risks of different anaesthetic choices for caesarean for a patient with a known cervical (C3) AVM that was stable throughout pregnancy [9]. They commented that using general anaesthetic might be particularly dangerous in the setting of a spinal AVM, as the patient may become hypertensive with increased intrathoracic and venous pressure on waking from the anaesthetic. This has potential to precipitate rupture of the AVM. Alternatively, perfusion of the spinal cord might be compromised by epidural anaesthesia as a result of hypotension and increased epidural pressure. They elected for spinal anaesthesia and delivered her baby via caesarean without complications. However, it would not be appropriate to use their study to inform anaesthetic choice for our patient because her AVM was much lower and had associated haemorrhage and as such, general anaesthetic was considered the safest option.

Treatment of spinal AVMs generally involves surgery, endovascular embolisation, or both [1, 2]. In two of the aforementioned cases, delivery alone facilitated neurological recovery as symptoms were caused by compression of vessels by the gravid uterus that was relieved after delivery [5, 6]. Unfortunately, our patient has shown no signs of neurological recovery following delivery, likely because her symptoms are as a result of an intramedullary lesion with associated haemorrhage.

Our patient's AVM had both intra- and extramedullary components. These lesions have complex angioarchitecture and as such are particularly difficult to treat [1]. Patsalides et al. proposed that a palliative approach to patients with such complex lesions is reasonable given that curative treatment would be “extremely difficult and likely associated with increased morbidity” [1].

A recent article published in 2017 by Rashad and colleagues in* Neurosurgical Review *described a novel method for treating intramedullary AVMs using stereotactic radiosurgery [10]. Of note, this technique was used in two patients who had suffered haemorrhages and were not suitable for surgery or embolisation. As such, they performed radiosurgery using CyberKnife™, a technique that uses targeted doses of radiation. For the two patients who had evidence of haemorrhage, one had improvement of their symptoms and one remained stable with no further haemorrhagic episodes.

Rehabilitation is an important aspect of treatment for patients with paraplegia. When a mother becomes paraplegic during pregnancy, it is essential to consider the newborn baby as well as the mother's ability to care for the baby after delivery. In our patient's case, she required a special exemption from the hospital executive to be considered for spinal rehabilitation with a newborn baby. Notably, her main concern when discussing discharge destinations was her desire to remain with her daughter.

While new onset paraplegia during pregnancy secondary to ruptured spinal AVM is very rare, it is important to discuss these cases to inform future practice. Timely recognition and appropriate management of these women might help prevent permanent disability.
